# Measuring stress in medical education: validation of the Korean version of the higher education stress inventory with medical students

**DOI:** 10.1186/s12909-016-0824-9

**Published:** 2016-11-24

**Authors:** Eun-Jung Shim, Hong Jin Jeon, Hana Kim, Kwang-Min Lee, Dooyoung Jung, Hae-Lim Noh, Myoung-Sun Roh, Bong-Jin Hahm

**Affiliations:** 1 Department of Psychology, Pusan National University, Busan, Republic of Korea; 2 Department of Psychiatry, Depression Center, Samsung Medical Center, Sungkyunkwan University School of Medicine, Seoul, Republic of Korea; 3 Department of Psychology, Ajou University, Suwon, Republic of Korea; 4 Department of Neuropsychiatry, Seoul National University Hospital, Seoul, Republic of Korea; 5 Department of Psychiatry and Behavioral Sciences, Seoul National University College of Medicine, Seoul, Republic of Korea; 6 Department of Human Factors Engineering, Ulsan National Institute of Science and Technology, Ulsan, Republic of Korea; 7 Health Service Center, Seoul National University, Seoul, Republic of Korea

**Keywords:** Higher education, Stress, Medical students, Measurement

## Abstract

**Background:**

Medical students face a variety of stressors associated with their education; if not promptly identified and adequately dealt with, it may bring about several negative consequences in terms of mental health and academic performance. This study examined psychometric properties of the Korean version of the Higher Education Stress Inventory (K-HESI).

**Methods:**

The reliability and validity of the K-HESI were examined in a large scale multi-site survey involving 7110 medical students. The K-HESI, Beck Depression Inventory (BDI) and questions regarding quality of life (QOL) and self-rated physical health (SPH) were administered.

**Results:**

Exploratory factor analysis of the K-HESI identified seven factors: Low commitment; financial concerns; teacher-student relationship; worries about future profession; non-supportive climate; workload; and dissatisfaction with education. A subsequent confirmatory factor analysis supported the 7-factor model. Internal consistency of the K-HESI was satisfactory (Cronbach’s α = .78). Convergent validity was demonstrated by its positive association with the BDI. Known group validity was supported by the K-HESI’s ability to detect significant differences on the overall and subscale scores of K-HESI according to different levels of QOL and SPH.

**Conclusions:**

The K-HESI is a psychometrically valid tool that comprehensively assesses various relevant stressors related to medical education. Evidence-based stress management in medical education empirically guided by the regular assessment of stress using reliable and valid measure is warranted.

## Background

Research with medical students consistently suggests the high burden of medical education. Medical students face a variety of stressors associated with their education; if not promptly identified and adequately dealt with, it may bring about several negative consequences in terms of mental health and academic performance.

In fact, medical students are more vulnerable to psychological distress and mental health problems than their non-medical peers [1]. Depression is one of the most highly prevalent mental health issues in medical students and it is associated with a higher likelihood of burnout, dropping out of medical school, and suicide [2–4]. It has also been found to be related to lower academic performance [5]. Research also suggested that 45–56% of medical students had symptoms suggestive of burnout [6]. Moreover, Pagnin and de Queiroz [7] compared the quality of life of 206 medical students with 199 peers in the general population and found that medical students reported lower psychological well-being and lower quality social relationships than their counterparts. Moreover, 50.5%, 44.2%, 28.7%, and 22.8% of students reported a low quality of life in psychological, social, physical health, and environment domains, respectively.

Early identification of stressors that students face is important for enabling interventions before these develop into more serious, aforementioned, consequences.

These studies identified several such factors associated with poor quality of life in medical students, such as social support [8], frustration with the curriculum [9], the teacher-student relationship [9], and financial difficulty [10]. Early identification of the stressors that medical students face in their education is important for enabling interventions before these develop into serious consequences, such as burnout or depression. Research suggests that 45% – 56% of medical students have symptoms suggestive of burnout [6], which has been found to be related to lower academic performance [5] and dropping out of medical school [4]. Depression has also been identified as one of the most prevalent mental health problems in medical students and it is associated with a higher likelihood of burnout and suicide [3, 11].

Despite the importance of systematic assessment of the stressors associated with higher education, there are few psychometrically validated assessment tools to measure stress in students enrolled in higher education, especially for those in medical programs. One such measure that does exist is the Higher Education Stress Inventory (HESI), developed by Dahlin, Joneborg and Runenson [12] in Sweden, which is partly based on the Perceived Medical School Stress Scale [13]. The strength of the HESI is that it assesses various stressful aspects of higher education. Major stressors in college students were categorized at the individual, dyadic, and group levels [14], and overall, the HESI comprehensively evaluates issues in these categories. Moreover, the HESI can be applied in a range of higher educational settings, allowing comparison among students in various disciplines. As factors from the HESI were significantly associated with depression [12], it might provide insights into preventive strategies for depression and related mental health issues in the post-secondary student population. But as its developers suggested, its validity has to be examined in more detail and in a larger population. Also, considering the fact stress factors in higher education might vary across different sociocultural contexts, its reliability and validity should be re-examined.

In view of this, the aim of the current study was to examine the psychometric properties of the Korean version of the HESI in a large representative sample of Korean medical students.

## Method

### Participants and procedure

The participants consisted of 7110 medical students in 41 medical schools in South Korea. Detailed information for participants and procedures are found in the previous study by Roh, Jeon, Kim, Han, & Hahm (2010) using the same dataset [5]. The mean age was 23.9 (*SD* = 2.65) years. The majority of participants were male (4427, 62.5%), identified as being religious (4053, 57.1%), and did not need to provide tuition and living expenses for themselves (5481, 77.3%). In terms of year of program, it was relatively well-distributed (1^st^ year: 2192, 31.1%; 2^nd^ year: 1982, 28.1%; 3^rd^ year: 1882, 26.7%; and 4^th^ year: 1003, 14.2%). This study was approved by the Institutional Review Board of the Seoul National University Hospital (C-0610–0207-186) and all participants provided written informed consent.

### Measures

#### Higher education stress inventory (HESI)

The HESI was developed to measure educational stress, primarily for medical students [12] but it can also be applied to various higher educational settings. It includes 33 items regarding the presence of stressors in a higher educational context. Respondents rate each statement on a 4-point Likert-type scale (1 = ‘does not apply at all’; 4 = ‘applies perfectly’). Factor analysis of the original HESI identified seven factors with 24 items, explaining 48.7% of the total variance. Cronbach’s α was .85 and the alpha values for the seven subscales are satisfactory: ‘worries about future endurance/competence (α = .78); non-supportive climate (α = .71); faculty shortcomings (α = .69); workload (α = .65); insufficient feedback (α = .65); low commitment (α = .62); financial concerns (α = .59).

The original English version of the questionnaire underwent a forward and backward translation (Korean↔English) procedure in line with the European Organization for the Research and Treatment of Cancer (EORTC) guidelines [15]. The forward translation was conducted by two independent clinicians (a psychiatrist and a psychologist). The research team examined, in detail, the two versions and finalized the version to be submitted for the backward translation. The backward translation was performed by a native English speaker with a good command of the Korean language, blinded to the original version of the HESI, to ensure semantic and structural equivalency.

### Beck depression inventory (BDI)

Depression was measured with the Korean version of the Beck Depression Inventory (K-BDI) [16, 17]. Scores on the 21-item self-rating scale of depression range from 0 to 63. Spearman-Brown coefficient and Cronbach’s alpha was .75 and .85 [17]. A total score of 10 to 15 is considered to be a mild level of depression, a score of 16-23 is a moderate level of depression, and a score of over 24 is considered to be a severe level depression.

### Quality of life and self-rated physical health

Students were asked to rate their quality of life (QOL) and self-rated physical health (SPH) on three levels (good, average and poor) in reference to their study period in medical school (How do you rate your quality of life during your study period in medical school?; How do you rate your physical health during your study period in medical school?).

Questions regarding socio-demographic factors such as age, gender, religion, and financial situation were also included in the survey.

### Statistical analyses

Internal consistency of the scale was examined using Cronbach's α. To examine construct validity, an exploratory factor analysis (EFA: principal axis factor analysis with promax rotation). A confirmatory factor analysis (CFA) was then performed on the same data set which served to rule out any methodological issues that may have identified an inappropriate factor result in the EFA [18]. The scree plot method and Kaiser criterion (Eigen value ≥ 1.0) were considered in determining the factor solution. Moreover, items with factor loadings < .30 on a single factor and factor loadings ≥ .30 on more than one factor were eliminated [19]. To assess goodness-of-fit indices of the 7-factor model of the K-HESI, several criteria were applied. Following current conventional recommendations, acceptable model fit was determined with standardized root mean square residual (SRMR) values of ≤ .08, root mean square error of approximation (RMSEA) values of ≤ .06 to .08,, and values approaching .95 for the Tucker-Lewis Index (TLI), Comparative Fit Index (CFI) and goodness-of-fit index (GFI) [20]. To examine convergent validity, Pearson correlations between scores of the K-HESI and the BDI were analyzed. To examine known-group validity, mean differences on the K-HESI subscale scores, across three levels of QOL and SPH, were examined using analysis of covariance (ANCOVA). Age, gender, year of program, religion, financial situation and depression scores were used as covariates. Statistical analyses were performed using SPSS 21.0 and AMOS 23.0 [21].

## Results

### Construct validity

The construct validity of the K-HESI was examined using exploratory and confirmatory factor analysis (EFA and CFA). The Kaiser-Meyer-Olkin measure of sampling adequacy was .84, above the recommended .50 cut-off value. Additionally, a Bartlett test for sphericity (*χ* ^2^_(528)_ = 552551.94, *p* < .000) confirmed the equality of variances across the samples.

Initial factor analysis identified 8 factors using Kaiser's criterion. To enhance the factor structure, factor loadings were examined and items 7 and 22 were removed due to low factor loadings (<.30). Four items (3, 5, 15, 21) having factor loadings of ≥ .30 on more than one factor without a clearly stronger loading on any specific factor were also removed. Items 18 and 24 (discrimination due to gender and ethnicity) were identified as the 8^th^ factor but the Cronbach’s alpha value was not optimal (α = .41) and explained only 1.17% of the total variance. Item 27 (Student union activities not promoting a sense of community) loaded on the third factor (teacher-student relationship, loading = .37) but it was difficult to explain as one dimension. Therefore, these three items (18, 24, 27) were excluded to enhance the factor structure of the K-HESI. Re-analysis was conducted with the remaining 24 items identified across seven factors. Results showed low extraction communalities for items 1 (studies control my life) and 13 (lack of support from peers) with values of .15 and .19 respectively, and exclusion of these two items improved internal consistency. The final result is shown in Table 1. A total of 45.8% of the variance was explained by the following factors: 1) low commitment - general dissatisfaction with major and curriculum; 2) financial concerns - worries about debt and financing; 3) teacher-student relationship - lack of feedback or respect from teacher; 4) worries about future profession - worries related to stress and workload in the future profession 5) non supportive climate - impersonal peer relationships; 6) workload - concerns about the amount and pace of the study load; and 7) dissatisfaction with education - issues related to educational activities.

**Table 1:** Factor categories and loadings* (*N* = 7110)

Factors and Items (Total Cronbach’s *α* = .78)		Factor loading						
		1	2	3	4	5	6	7
1. Low Commitment (4items); *α* = .78; *M*(*SD*) = 1.99(0.51)								
17.	Not proud of profession	**.783**	.132	.178	.083	.029	-.008	.188
10.	Not satisfied with choice of career	**.757**	.096	.220	.160	.071	.076	.162
6.	Personal development not stimulated through studies	**.599**	.085	.322	.050	.121	.106	.177
26.	Sense of education not giving adequate preparation for profession	**.594**	.108	.164	-.008	.042	-.047	.217
2. Financial Concerns (3items); *α* = .77; *M*(*SD*) = 2.15(0.71)								
12.	Worries over financing during education	.069	**.777**	.054	.167	.209	.113	.261
28.	Worries over future economy (debts from studies)	.152	**.770**	.034	.207	.166	.119	.341
23.	Worries about housing	.119	**.652**	-.026	.144	.148	.057	.302
3. Teacher-Student Relationship (4items); *α* = .69; *M*(*SD*) = 2.73(0.52)								
8.	Lack of encouragement from teachers	.189	.013	**.723**	.106	.122	.189	.095
2	Lack of respectful treatment from teacher	.313	.090	**.655**	.117	.186	.213	.255
19.	Lacking opportunities for influencing studies or curriculum	.073	-.010	**.573**	.126	.150	.238	.090
33.	Lack of feedback from teachers	.230	.009	**.462**	.017	.134	.096	.158
4. Worries about Future (2items); *α* = .82; *M*(*SD*) = 2.96(0.72)								
14.	Worries over workload in the future	.094	.204	.131	**.852**	.259	.329	.262
20.	Worries over stress in future profession	.101	.201	.131	**.810**	.285	.369	.339
5. Non-supportive Climate (3items); *α* = .65; *M*(*SD*) = 2.74(0.60)								
11.	Cold and impersonal attitude enhanced by education	.049	.153	.168	.223	**.733**	.326	.335
9.	Competitive attitude among students	.016	.126	.136	.188	**.627**	.278	.267
4.	Anonymity among students	.131	.200	.150	.209	**.496**	.209	.289
6. Workload(2items); *α* = .76; *M*(*SD*) = 3.08(0.63)								
30.	Literature too difficult and extensive	.032	.101	.237	.355	.332	**.860**	.261
31.	Pace of studies too high	.022	.101	.247	.289	.349	**.711**	.267
7. Dissatisfaction with educational activities (4items); *α* = .53; *M*(*SD*) = 2.49(0.46)								
32.	Must attend situation that are ethically offending	.167	.216	.095	.103	.203	.110	**.563**
29.	Too much student-controlled group-activities, resulting in unclear curriculum	.158	.255	.175	.220	.332	.347	**.485**
16.	Unclear role and function as student	.028	.144	.120	.291	.226	.205	**.418**
25.	Perceiving many future colleagues as dissatisfied or dejected in their profession	.150	.242	.121	.307	.273	.214	**.414**
Eigenvalue		3.388	1.964	1.591	1.023	0.847	0.807	0.452
of variance %		15.399	8.927	7.232	4.650	3.850	3.667	2.055
Cumulative %		15.399	24.326	31.558	36.208	40.057	43.724	45.780

Note. Boldface indicates highest factor loadings

*Principal axis analysis with promax rotation with Kaiser Normalization, rotation converged in 6 iterations

Regarding correlations among the subscales of the HESI (Table 3), the 7^th^ factor, dissatisfaction with education, showed overall higher associations with other factors such as financial concerns (*r* = .29**), worries about future (*r* = . 33**), non-supportive climate (*r* = .33**), and workload (*r* = .29**). Correlations of workload with worries about future (*r* = .33**) and non-supportive climate (*r* = .30**) were also higher.

Internal consistency of the K-HESI was satisfactory (α = 0.78 for the total scale; range of α for subscales = .53 – .82) (Table 1).

Additionally, the CFA results indicate that the 22-item seven-factor model of the K-HESI, compared to the original 24-item seven-factor model, produced a better fit of indices for Korean medical students (Table 2). The *χ* ^2^ values for both models were significant. However, the *χ* ^2^ test is considered to be extremely sensitive to sample size, with larger sample sizes, relatively small discrepancies between the observed data matrix and the predicted matrix can produce significant *χ* ^2^ values [22]. The examination of other indices, such as SRMR, TLI, CFI and RMSEA, indicate that the K-HESI’s factor structure fit the data of the Korean medical students better than the original factor structure suggested by the original Swedish study [12].

**Table 2:** Result of goodness-of-fit indices of two models (*N =* 7110)

Models	*x* ^*2*^	*DF*	*p*	*GFI*	*SRMR*	*TLI*	*CFI*	*RMSEA* *[LO90, HI90]*
Original HESI	8517.64	231	.00	.897	.076	.739	.781	.071 [.070-.072]
K-HESI	2667.85	188	.00	.966	.038	.920	.935	.043[.042-.045]

### Convergent validity

Convergent validity of the K-HESI was demonstrated by its positive correlations with BDI scores (Table 3). The K-HESI total score and all subscales scores were significantly associated with BDI scores (*r* = .42** with the total HESI). Correlations of the subscales of low commitment(*r* = .30**), teacher-student relationship (*r* = .26**), and non-supportive climate (*r* = .26**) with the BDI were relatively higher.

**Table 3:** Correlations of the K-HESI and BDI

	1	2	3	4	5	6	7	8
1. HESI total	-							
2. Low Commitment	.498**	-						
3. Financial Concerns	.525**	.112**	-					
4. Teacher-student relationship	.537**	.258**	.021	-				
5. Worries about future	.539**	.096**	.189**	.119**	-			
6. Non supportive Climate	.582**	.080**	.181**	.166**	.246**	-		
7. Workload	.509**	.031**	.092**	.224**	.325**	.302**	-	
8. Dissatisfaction with education	.663**	.167**	.291**	.176**	.325**	.331**	.287**	-
9. BDI Depression	.424**	.296**	.194**	.259**	.211**	.257**	.224**	.209**

**p <. 01

### Known-group validity of the HESI in terms of quality of life and self-rated physical health

The results of the ANCOVA to examine known-group validity of the K-HESI across three levels of quality of life (QOL) and self-rated physical health (SPH) are shown in Tables 4 and 5. Regarding the QOL, the K-HESI total and subscale scores were the highest in the poor QOL group followed by those with average and good level of QOL group with the modest effect size (*η* ^*2*^ *=* .09). As for the SPH, the scores showed a significant difference across three levels of SPH with small effect size (*η* ^*2*^ *=* .03).

**Table 4:** K-HESI subscale scores according to the level of quality of life

	Quality of life (*N* = 6965)			*F*	*η* ^*2*^	*Post hoc*
Variables	Poor^a^ (*n* = 2673)	Average^b^ (*n* = 3160)	Good^c^ (*n* = 1132)			
	*M(SD)*	*M(SD)*	*M(SD)*			
HESI Total^d^	2.67(.31)	2.47(.27)	2.33(.31)	308.40***	.085	c < b < a
Low Commitment	2.09(.51)	1.93(.42)	1.71(.46)	134.13***	.039	c < b < a
Financial Concerns	2.25(.75)	2.13(.67)	2.00(.68)	10.51***	.003	c < b, a
Teacher-student relationship	2.91(.52)	2.68(.47)	2.53(.51)	134.53***	.039	c < b < a
Worries about future	3.17(.57)	2.88(.66)	2.70(.77)	112.36***	.033	c < b < a
Non-Supportive Climate	2.93(.61)	2.65(.55)	2.54(.59)	111.54***	.033	c < b < a
Workload	3.28(.61)	2.99(.59)	2.85(.66)	113.87***	.033	c < b < a
Dissatisfaction with education	2.59(.48)	2.44(.43)	2.38(.47)	45.21***	.013	c < b < a

^a^ Poor; ^b^ Average; ^c^ Good; ^d^ K-HESI Item scores range from 1(does not apply at all) to 4(applies perfectly)

***p < .000

**Table 5:** HESI scores according to the level of self-rated physical health

	Self-rated physical health (*N* = 6644)			*F*	*η* ^*2*^	*Post hoc*
Variables	Poor^a^ (*n* = 1236)	Average^b^ (*n* = 3213)	Good^c^ (*n* = 2201)			
	*M(SD)*	*M(SD)*	*M(SD)*			
HESI Total^d^	2.68(.32)	2.53(.29)	2.42(.31)	84.09***	.025	c < b < a
Low commitment	2.13(.54)	1.97(.45)	1.82(.45)	78.52***	.023	c < b < a
Financial concern	2.29(.75)	2.18(.69)	2.01(.69)	15.95***	.005	c < b < a
Teacher-student relationship	2.90(.53)	2.74(.49)	2.66(.51)	21.97***	.007	c < b < a
Worries about future	3.13(.73)	2.98(.68)	2.85(.74)	18.63***	.006	c < b < a
Non-supportive Climate	2.94(.62)	2.73(.57)	2.63(.60)	26.63***	.008	c < b < a
Workload	3.27(.65)	3.08(.59)	2.98(.65)	17.98***	.005	c < b < a
Dissatisfaction with education	2.60(.48)	2.48(.44)	2.43(.46)	17.45***	.005	c < b < a

^a^ Poor; ^b^ Average; ^c^ Good; ^d^ K-HESI Item scores range from 1(does not apply at all) to 4(applies perfectly)

***p < .000

## Discussion

Current study examined the psychometric properties of the K-HESI and results supported its reliability and validity. Exploratory factor analysis of the K-HESI identified seven factors, which demonstrated good model fit in the CFA. Internal consistency was also satisfactory for the overall scale and its subscales. The 7-factor structure of the K-HESI was equivalent to that of the original measure; however, items within the factors varied, as did the relative importance of factors to the construct of higher educational stress [12]. Whereas in the original HESI scale, ‘worries about future endurance/competence’ was the factor which explained most of the higher educational stress, in the Korean sample, the factor of ‘low commitment’ , which includes items about general dissatisfaction with major and curriculum, was the strongest factor. Efforts to improve the low commitment of students will also be important because a high satisfaction with overall learning environment, which encompasses aspects of the “low commitment” factor, was independently associated with resiliency and recovery from burnout [2]. Similarly, in a qualitative investigation with medical students, frustration with the study program was one of the factors that was found to decreased quality of life [9]. The choice of a major in medicine is often guided by excellent academic grades in high school, or from the consideration of medicine as a profession assuring money and status in society [9], rather than the aptitude or interests of the individual student. This might have partly contributed to the low-commitment in medical education. In relation to this, a previous study with college students indicated that a perceived fit with an academic major was strongly associated with affective commitment to major and academic self-efficacy [23].

Moreover, while a non-supportive environment explained the second largest amount of variance in Swedish students, financial concerns were the second most influential factor in Korean students. In contrast, financial concerns explained the least amount of variance in the Swedish sample. Varying levels of economic burden associated with higher education in each country might explain this discrepancy as Korea is a country with a high economic burden associated with education [24, 25]. Furthermore, financial concerns were closely associated with dissatisfaction with education. This close association might reflect the economic burden felt by students which might affect the perceived level of satisfaction with the education they are receiving. A previous study found that 37.4% of students thought that worrying about money affected their studies and those who worried about money performed less well on examinations than their peers [26]. Similarly, previous research found that students who studied while holding a part-time job experienced more study-related problems and were more likely to report not having enough time, which led to sleep problems [27]. Financial difficulty is also one of the risk factors for major depressive disorder [5], suggesting the need to address these concerns in medical students.

The third factor, ‘teacher-student relationship’ includes issues related to perceived interactions with teachers. In the Swedish study, items regarding this issue were divided into two factors (faculty shortcomings and low commitment), but in the Korean study it emerged as one factor and seemed to represent a more important factor to Korean students, explaining 7.2% of the variance. This difference might be a reflection of the more collectivistic culture of Asia, in which students are more motivated by social approval, including positive feedback or recognition from authority figures, such as teachers [28]. In addition, previous findings suggest that the amount of support from faculty has a strong negative correlation to student burnout among 1^st^ and 2^nd^ year students [11]. The students’ perceptions of faculty members’ greater interest in their education was associated with a greater likelihood of resilience to, and recovery from, burnout [2]. Furthermore, meaningful relationships with teachers was found to increase quality of life in a qualitative study with Brazilian medical students [9]. Given that the ‘teacher-student relationship’ factor was also significantly associated with ‘low commitment’ , efforts to enhance the relationship between faculty members and students will be needed.

It is also worthy of note that the first factor in the Swedish study, ‘worries about future endurance/competence’ (explaining 19.5% of the variance), was only the fourth largest factor in this sample, explaining 4.65% of the variance in Korean students. The fact that worries related to the stress and workload in a future profession are more strongly related to overall stress in the Swedish sample, compared to the Korean sample, reflects probable differences in perspective towards one’s job between the two countries.

Convergent validity was supported by significant positive associations of the K-HESI with the BDI, consistent with the original HESI study [12]. These findings are also in line with previous research with German medical students in which perceived medical school stress was associated with depression and burnout [29]. The association of the low commitment, teacher-student relationship, and non-supportive climate factors with depression was more strongly related to burnout and depression compared to the other factors, suggesting a particular need for monitoring these factors in the prevention of depression in medical students.

Known-group validity was demonstrated by the ability of the overall K-HESI and its subscales to significantly detect different levels of QOL and SPH. Medical students who rated their quality of life and physical health as poor reported more elevated levels of higher education stress. However, the effect size of HES was small with respect to SPH.

The study does have some weaknesses that are worth noting. Due to the cross-sectional nature of the data collection, the test-retest reliability of the K-HESI could not be examined. Although stress level varies across time, the ability of the tool to detect the effects of interventions should be investigated in a longitudinal study. Another limitation was that convergent validity was examined only in relation to the BDI. Also, we did not use standardized measures to assess quality of life and self-rated physical health.

## Conclusions

This study explored various aspects of the stress associated with medical education. Using multi-site surveys and a representative sample of medical students in Korea, a significant influence of medical students’ higher education stress was confirmed, suggesting the need for an intervention to alleviate higher education stress. Moreover, although the relative importance of the factors comprising the K-HESI was found to have some differences to previous research using a Swedish sample, the current findings suggest a universal nature of higher education stress across countries.

To conclude, the K-HESI is a psychometrically valid tool that comprehensively assesses various relevant stressors related to higher education. Evidence-based stress management in medical education empirically guided by the regular assessment of stress using reliable and valid measure is warranted.

## Abbreviations

K-BDI: Korean version of Beck Depression Inventory
K-HESI: Korean version of Higher Education Stress Inventory
QOL: Quality of Life
SPH: Self-rated physical health
